# Suppression of FOXO1 is responsible for a growth regulatory repressive transcriptional sub-signature of EWS-FLI1 in Ewing sarcoma

**DOI:** 10.1038/onc.2013.361

**Published:** 2013-09-02

**Authors:** S Niedan, M Kauer, D N T Aryee, R Kofler, R Schwentner, A Meier, U Pötschger, U Kontny, H Kovar

**Affiliations:** 1 Children's Cancer Research Institute, St Anna Kinderkrebsforschung, Vienna, Austria; 2 Department of Pediatrics, Medical University, Vienna, Austria; 3 Division of Molecular Pathophysiology, Biocenter, Medical University Innsbruck, Innsbruck, Austria; 4 Division of Pediatric Hematology and Oncology, Department of Pediatrics and Adolescent Medicine, University Medical Center, Freiburg, Germany

**Keywords:** CDK2, Ewing sarcoma, EWS-FLI1, FOXO1

## Abstract

The Ewing sarcoma (ES) EWS-FLI1 chimeric oncoprotein is a prototypic aberrant ETS transcription factor with activating and repressive regulatory functions. We report that EWS-FLI1-repressed promoters are enriched in forkhead box (FOX) recognition motifs, and identify FOXO1 as a EWS-FLI1-suppressed regulator orchestrating a major subset of EWS-FLI1-repressed genes. In addition to FOXO1 regulation by direct promoter binding of EWS-FLI1, its subcellular localization and activity is regulated by cyclin-dependent kinase 2- and AKT-mediated phosphorylation downstream of EWS-FLI1. Restoration of nuclear FOXO1 expression in ES cells impaired proliferation and significantly reduced clonogenicity. Gene-expression profiling revealed a significant overlap between EWS-FLI1-repressed and FOXO1-activated genes. As a proof of principle for a potential therapeutic application of our findings, the treatment of ES cell lines with methylseleninic acid (MSA) reactivated endogenous FOXO1 in the presence of EWS-FLI1 in a dose- and time-dependent manner and induced massive cell death dependent on FOXO1. In an orthotopic xenograft mouse model, MSA increased FOXO1 expression in the tumor paralleled by a significant decrease in ES tumor growth. FOXO1 reactivation by small molecules may therefore serve as a promising strategy for a future ES-specific therapy.

## Introduction

Ewing sarcoma (ES) is characterized by aberrant activation of ETS transcription factors due to rearrangement with the *EWS* gene. The most common gene fusion combines *EWS* with *FLI1*,^1^ which has a rate-limiting role in ES pathogenesis,^2^ as interference with EWS-FLI1 expression or function results in tumor regression *in vivo* and dysregulates ES tumor cell growth *in vitro*.^3^

Although experimental evidence suggests that the EWS domain confers transcription activating properties to EWS-FLI1,^4^ knockdown of EWS-FLI1 uncovers similar numbers of repressed and activated genes.^5, 6, 7^ We focused on the repressive EWS-FLI1 regulatory network as the mechanisms and mediators of transcriptional repression downstream of EWS-FLI1 are poorly understood. We identified the enrichment of forkhead box (FOX) recognition motifs in EWS-FLI1-repressed genes. FOX transcription factors are a superfamily of more than 100 structurally related proteins with a conserved 100-residue DNA-binding domain, the forkhead domain. In humans, 19 subgroups named FOXA–FOXS have been discovered.^8^ The O-class includes FOXO1, FOXO3, FOXO4 and FOXO6.^9^ Importantly, FOXO proteins function as tumor suppressors by their capacity to induce cell-cycle arrest,^10^ apoptosis and DNA repair^11^ as well as modulate the expression of genes responsive to oxidative stress and cell differentiation.^12^ FOXO proteins are regulated by post-translational modifications involving phosphorylation, acetylation and ubiquitinylation.^13^ Subcellular localization and DNA-binding ability of FOXO transcription factors are regulated in response to external and internal stimuli by protein kinases such as AKT and cyclin-dependent kinase 2 (CDK2).^12^

Integrating expression data and binding-motif preferences, we identify FOXO1 repression as a growth limiting regulatory pathway downstream of EWS-FLI1 and as a potential target in ES. Here, we report that a major subset of the EWS-FLI1 repressive signature is comprised by FOXO1 target genes. We identify several layers of FOXO1 dysregulation by EWS-FLI1 and provide evidence that therapeutic reactivation of FOXO1 interferes with ES growth *in vitro* and *in vivo*.

## Results

### EWS-FLI1-repressed genes show enrichment of FOX recognition motifs

**Figure 1:**
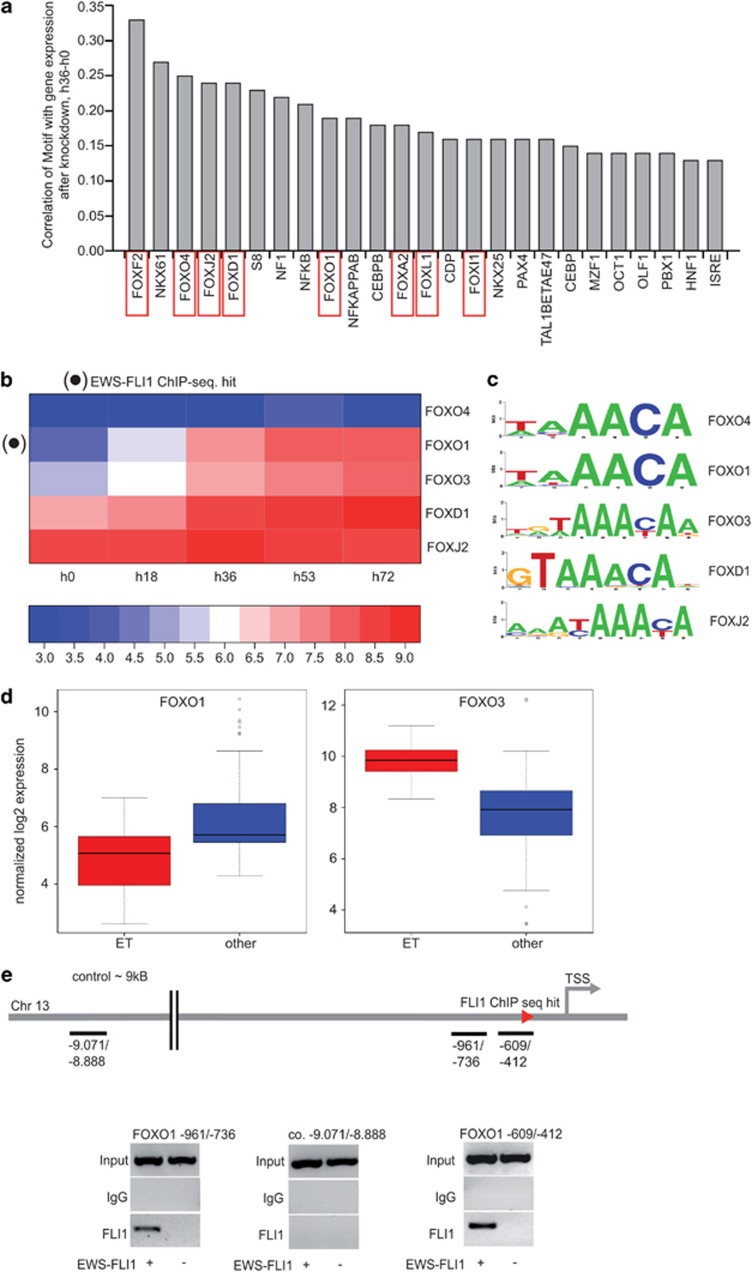
FOX motifs are enriched in EWS-FLI1-repressed genes, and FOXO1 and FOXO3 are transcriptionally repressed by EWS-FLI1. (**a**) Result from the correlation analysis of gene expression with motif occurence. *x* axis: DNA motifs from TRANSFAC; *y* axis: Pearson correlation of gene expression change at time point 36 h (against 0 h) of conditional EWS-FLI1 suppression in A673sh cells with the number of motifs. Forkhead box motifs are marked by red squares. (**b**) Time-resolved expression of FOX genes upon knockdown of EWS-FLI1 in A673sh cells. Only genes with probe sets that passed quality filtering (see Materials and methods section) are shown. (**c**) Sequence of DNA motifs predicted to be specifically recognized by individual A673sh expressed FOX-factors. (**d**) Box plot of normalized gene expression values for FOXO1 and FOXO3 in primary tumor tissue. Data taken from.^6^ Blue: reference tissues from.^49^ Red: primary ES samples. The comparison of FOXO1 and FOXO3 shows that FOXO1 is ‘off' in comparison with a wide variety of reference tissues, whereas FOXO3 is ‘on'. (**e**) Representative chromatin immunopreciptation (ChIP)-PCRs in A673sh cells on two different FOXO1 promoter fragments. Fragments range from −961 to −736 and from −609 to −412 upstream of the transcription start site, including the ChIP-Seq hit at position −534 to −298, respectively and, for control, from −9071 to −8888 further upstream. ETS-binding sites (GGAA core motif)^6^ within these regions were identified using the ConSite tool.^50^ Specific ETS motifs responsible for FLI1 binding are ACGGAAG for fragment (−609/−412) and TAGGAAG/CGGGAAG for fragment (−961/−736), respectively. Signals for EWS-FLI1 binding were obtained exclusively for the two promoter fragments in the presence of EWS-FLI1, but were completely abrogated upon 48 h of doxycycline-induced knockdown of EWS-FLI1. Input DNA and ChIPs using an immunoglobulin G (IgG) control antibody were used for specificity control.

To elucidate the transcriptional network downstream of EWS-FLI1, we analyzed time-resolved gene-expression profiles upon conditional EWS-FLI1 knockdown in A673sh cells. Promoter regions of knockdown-responsive genes were analyzed for the presence of transcription factor-binding motifs. Whereas EWS-FLI1-activated promoters (suppressed upon modulation of EWS-FLI1) revealed strong enrichment of ETS-binding motifs, EWS-FLI1-repressed gene promoters (activated upon EWS-FLI1 silencing) showed prominent over-representation of recognition motifs for FOX transcription factors while lacking enrichment of ETS motifs (Figure 1a). This result is consistent with a model where EWS-FLI1 mainly acts as a direct transcriptional activator, and suggests a role for FOX family members in EWS-FLI1-mediated gene repression.

### Several FOX proteins, including FOXO1 and FOXO3, are regulated by EWS-FLI1 at the transcriptional level

The enrichment of FOX motifs within EWS-FLI1-repressed promoters prompted us to investigate which FOX candidates are *per se* regulated by EWS-FLI1 in ES. Inspection of expression data of the inducible EWS-FLI1 knockdown as well as of five additional ES cell lines with transient EWS-FLI1 knockdown (previously described^6^) revealed consistently differential expression of FOXO1 and of the related FOXO3 between control and EWS-FLI1 knockdown conditions (Figure 1b and Kauer *et al.*^6^). Furthermore, FOXO1 is expressed at lower levels in primary ES compared with a wide variety of tissues^14^ (Figure 1d), and its promoter is directly bound by EWS-FLI1 in chromatin immunopreciptation-PCR experiments (Figure 1e).^15^ The FOXO3 promoter, though, was not directly bound by EWS-FLI1 (data not shown) and shows higher expression in ES than in most other tissues (Figure 1d). Finally, position weight matrices (sequence logos, Figure 1c) of ES expressed FOX-factors were found to be very similar. Therefore, combining the motif analysis with gene expression data, FOXO1 and FOXO3 emerged as candidate regulatory transcription factors for a repressive transcriptional sub-signature downstream of EWS-FLI1.

### Subcellular localization of FOXO1 is regulated by EWS-FLI1

**Figure 2:**
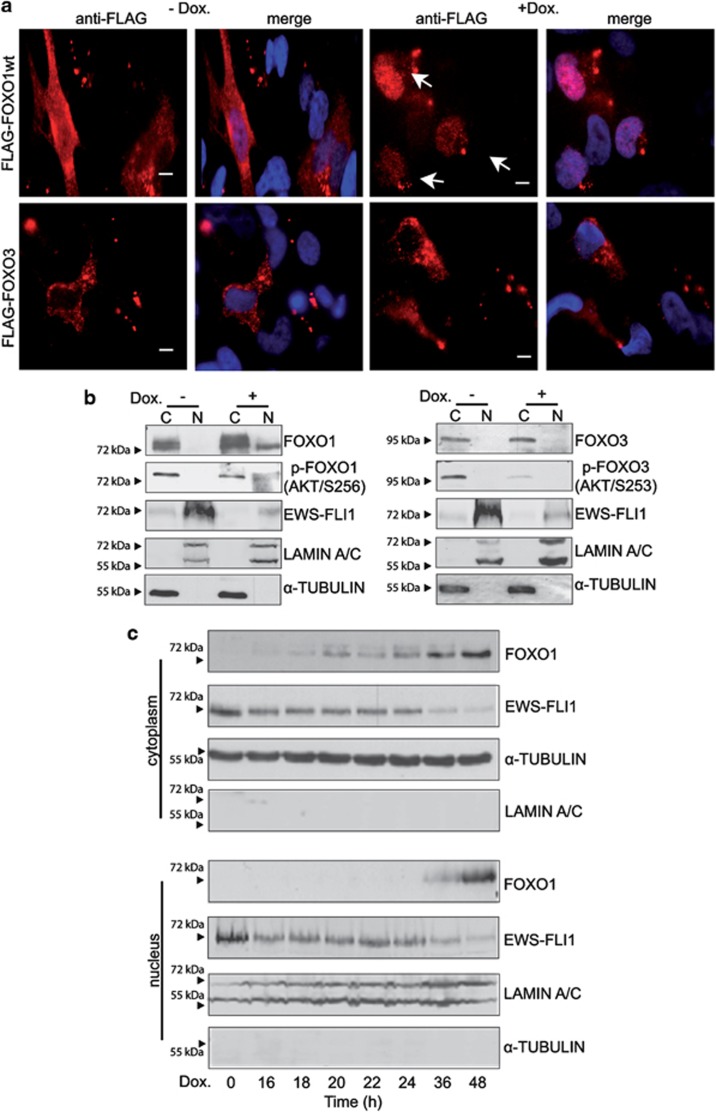
Subcellular localization of FOXO1 in A673sh cells is dependent on EWS-FLI1. (**a**) Immunofluorescence analysis of FOXO1 and FOXO3 subcellular localization in the presence or absence of EWS-FLI1. Flag-tagged FOXO1 or FOXO3 was transfected into A673sh cells in the absence and presence of doxycycline-induced EWS-FLI1 knockdown for 48 h post transfection, and subcellular localization was monitored by immunofluorescence using FLAG-tag-specific antibody (red) with 4',6-diamidino-2-phenylindole counterstain to delineate cell nuclei (blue). In the presence of EWS-FLI1, FOXO1 and FOXO3 stainings were confined exclusively to the cytoplasm. Upon EWS-FLI1 knockdown, only ectopically expressed FOXO1 but not FOXO3 translocated to the nucleus. (**b**) Immunoblot analysis of endogenous FOXO1 expression in cytoplasmic and nuclear cell fractions of A673sh cells. Basal expression of endogenous FOXO1 and FOXO3 in the presence of EWS-FLI1 was detectable exclusively in the cytoplasm. However, upon knockdown of EWS-FLI1 by doxycycline treatment for 48 h, FOXO1 expression was significantly increased and also expressed in the nucleus, whereas FOXO3 remained in the cytoplasm. In concordance with this observation, the levels of inactive phosphorylated FOXO1 and FOXO3 were reduced upon knockdown of EWS-FLI1. Note, that the double-band-appearance of FOXO1 is due to unspecific antibody recognition of both phosphorylated (upper) and unphosphorylated (lower) FOXO1 protein. (**c**) Kinetics of endogenous FOXO1 protein expression upon doxycycline-induced EWS-FLI1 knockdown in A673sh cells. Downregulation of EWS-FLI1 was already observed at 16 h, the time when cytoplasmic FOXO1 induction became first detectable, whereas significant nuclear FOXO1 expression was first observed at 36 h of doxycycline treatment with strongest expression at 48 h. Scale bars: 10 μM.

We next tested the subcellular localization of FOXO1 and FOXO3 when expressed in A673sh cells. In the presence of EWS-FLI1, both ectopic Flag-FOXO1 and Flag-FOXO3 were found excluded from the nucleus. Upon the silencing of EWS-FLI1, however, Flag-FOXO1 but not Flag-FOXO3 readily translocated to the nucleus (Figure 2a). We also monitored endogenous FOXO1 and FOXO3 proteins by immunoblotting and observed strong FOXO1 induction and only weakly increased FOXO3 levels upon EWS-FLI1 knockdown. FOXO1 was confined to the cytoplasm and the nucleus, whereas FOXO3 was exclusively retained in the cytoplasm (Figure 2b). Although the cytoplasmic increase in FOXO1 expression was already detectable at 16 h of doxycycline treatment (the earliest time point of EWS-FLI1 knockdown), nuclear FOXO1 was first observed at 36 h (Figure 2c). Our findings suggest that EWS-FLI1 regulates FOXO1 not only at the transcriptional level but also affects its subcellular localization thereby controlling FOXO1 transcriptional activity.

### FOXO1 is tightly regulated by CDK2- and AKT-mediated phosphorylation in ES

**Figure 3:**
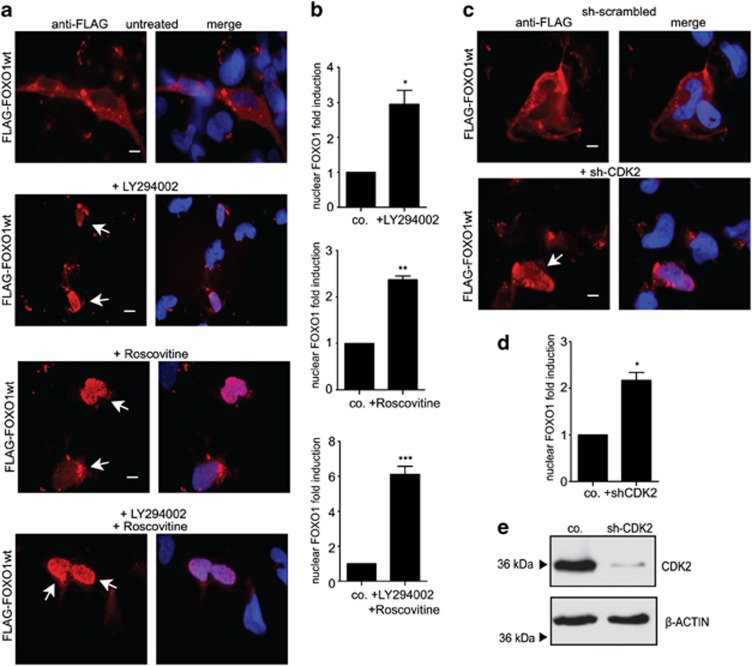
Nuclear localization of FOXO1 but not FOXO3 is tightly regulated by AKT and CDK2. (**a**) Representative immunofluorescence analysis of A673sh cells transfected with Flag-FOXO1wt and treated or not with 40 μM of the PI3K/AKT inhibitor LY294002 or 40 μM of the canonical CDK2 inhibitor roscovitine or a combination of both 48 h post transfection for 24 h. Roscovitine and LY294002 treatments alone or in combination restore FOXO1 nuclear localization in the presence of EWS-FLI1, whereas FOXO3 remains in the cytoplasm (Supplementary Figures 2a and b). Results are representative of three experiments. (**b**) Quantification of exogenously expressed Flag-FOXO1 in nuclear cell fractions using LICOR Odyssey Infrared Imaging System (**c, d**) Knockdown of endogenous CDK2 by shRNA-mediated RNA interference is sufficient to restore nuclear FOXO1 localization. A673sh cells were transfected with FLAG-FOXO1 and either shCDK2 or a scrambled shRNA control, and immunofluorescence analysis (**c**) or cell fractionation followed by western blotting and quantification using LICOR Odyssey Infrared Imaging System (**d**) was conducted 48 h post transfection. (**e**) Efficient CDK2 protein knockdown was monitored on the immunoblot. Results for quantification of nuclear FOXO1 represent the mean±s.e.m. of three experiments performed. *P<0.05, **P<0.01 and ***P<0.001. Scale bars: 10 μM.

The subcellular localization of FOXO1 and FOXO3 transcription factors is known to be mainly regulated by post-translational phosphorylation.^12^ Mining the gene expression data upon EWS-FLI1 knockdown in A673sh cells and in five additional ES cell lines,^6^ and of our EWS-FLI1 chromatin immunoprecipitation-seq data, we identified CDK2, a well-known negative regulator of FOXO1 transcriptional activity,^16^ among EWS-FLI1-induced genes^6^ (Supplementary Figure 1). In addition, it has been reported that PI3K/AKT-mediated phosphorylation negatively regulates FOXO1/3 nuclear localization and consequently activity.^11^ To investigate whether CDK2 and/or AKT have a role in nuclear exclusion of FOXO1/3 downstream of EWS-FLI1, we treated A673sh cells with the inhibitors roscovitine and LY294002 alone, or in combination, to inhibit CDK2 and AKT activity, respectively (Figure 3a and Supplementary Figure 2a). At concentrations that did not induce toxicity but only mild signs of apoptosis, both inhibitors significantly restored nuclear localization of ectopically expressed Flag-FOXO1 (Figures 3a and b), whereas Flag-FOXO3 remained in the cytoplasm (Supplementary Figures 2a and b). Coupled with the absence of nuclear translocation upon EWS-FLI1 knockdown, this result made FOXO3 unlikely to participate in the gene regulatory network downstream of EWS-FLI1.

To test the importance of CDK2- and AKT-mediated phosphorylation for the transcriptional activity of FOXO1, we transiently transfected A673sh cells with phosphorylation-resistant FOXO1 mutants. We used Flag-FOXO1-T24A/S256A/S319A (Flag-FOXO1-AAA) and Flag-FOXO1-S249A/S298A, in which AKT, respectively, CDK2-specific phosphorylation sites were converted to alanine.^16, 17^ Both FOXO1 mutants localized to the nucleus (Supplementary Figure 2c). As treatment with roscovitine or mutation of residues S249 and S298 was sufficient to restore nuclear FOXO1 expression, we hypothesized that CDK2 had a central role in FOXO1 regulation downstream of EWS-FLI1. To test this, we performed knockdown experiments targeting endogenous CDK2 with specific short hairpin RNA (shRNA) in the presence of EWS-FLI1. As shown in Figures 3c–e, transiently transfected Flag-FOXO1wt was significantly expressed higher in the nucleus when endogenous CDK2 was concomitantly silenced. No influence on subcellular FOXO1 localization was observed using a scrambled shRNA control. These results confirm a rate-limiting role for CDK2 in nuclear FOXO1 exclusion in EWS-FLI1-expressing ES cells.

### A repressive sub-signature of EWS-FLI1 regulated genes is due to suppression of FOXO1

**Figure 4:**
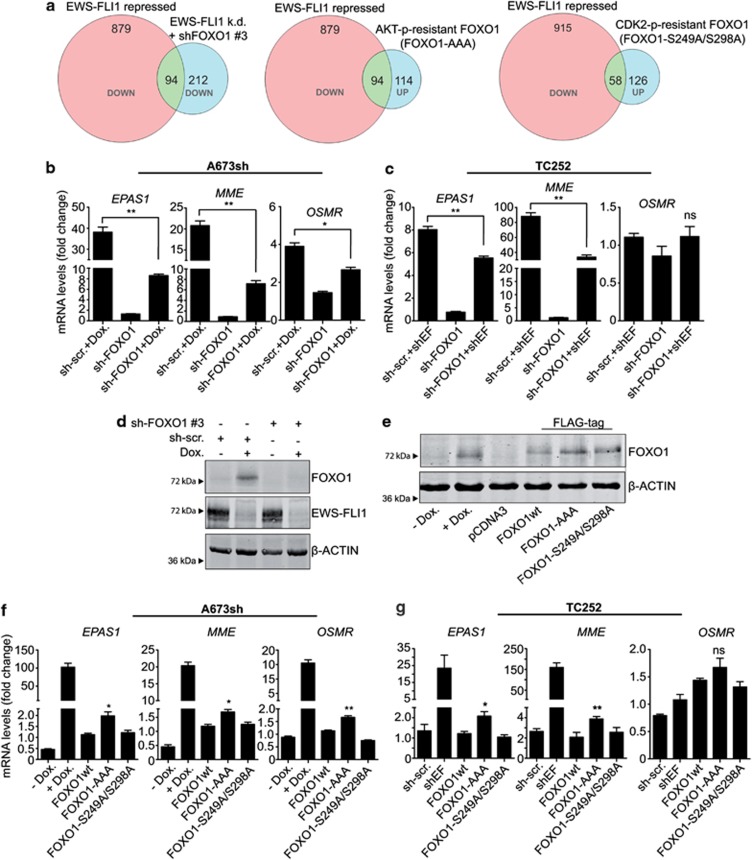
A subset of EWS-FLI1-repressed genes is regulated by FOXO1. (**a**) Venn Diagram showing the intersection between EWS-FLI1-repressed and FOXO1-activated genes. Silencing of reactivated endogenous FOXO1 under EWS-FLI1 knockdown conditions prohibited the activation of 94 EWS-FLI1-repressed genes, whereas the overexpression of nuclear FOXO1 mutants (Flag-FOXO-T24A/S256A/S319A(AAA) (AKT-p-resistant), Flag-FOXO-S249A/S29A (CDK2-p-resistant) led to reactivation of 94 and 58 EWS-FLI1-repressed genes, respectively. (**b, c**) Knockdown of reactivated endogenous FOXO1 was used to demonstrate the dependency of EWS-FLI1-mediated repression on FOXO1 suppression in two different ES cell lines. The mRNA expression was normalized to pCDNA3 empty vector control expression or to sh-scrambled control without doxycycline treatment or sh-RNA targeting EWS-FLI1 (shEF), and statistical relevance was analyzed using the unpaired *t*-test or one-sample *t*-test, respectively. B2M was used as internal control and doxycycline was applied for 72 h. (**d**) Representative FOXO1 protein expression corresponding to **b**, showing specificity and efficiency of sh-RNA-mediated FOXO1 silencing (shRNA no. 3). β-Actin was used as loading control. (**e**) Representative western blot for protein expression of endogenous FOXO1 upon inducible EWS-FLI1 knockdown as well as ectopically expressed wild-type and nuclear FOXO1 mutant, corresponding to (**f**) showing similar levels of ectopically expressed and induced endogenous FOXO1. (**f, g**) Validation of EWS-FLI1-repressed genes that can be reactivated by nuclear FOXO1 in two ES cell lines. A673sh and TC252 cells were transfected with AKT- or CDK2-phosphorylation-resistant versions of Flag-FOXO1, and mRNA expression of three genes representative of the overlap between EWS-FLI1-repressed and FOXO1-activated genes was measured by reverse transcription–quantitative PCR (RT-qPCR). Results represent the mean±s.e.m. of three experiments performed. *P<0.05 and **P<0.01. *EPAS1*, endothelial PAS domain protein 1 (entrez gene number: ID: 2034); *MME*, membrane metallo-endopeptidase (ID: 4311); *OSMR*, oncostatin M receptor (ID: 9180)

To test for the contribution of FOXO1 inactivation to the EWS-FLI1 repressive transcriptional signature, the overlap between genes reactivated upon EWS-FLI1 knockdown and (i) genes rescued from the reactivation after combined EWS-FLI1 and FOXO1 silencing and (ii) genes activated by ectopically expressed, nuclear directed FOXO1 were determined (Figure 4a). In these experiments, 973 genes were found significantly (logFC>1, *P*<0.05) activated after EWS-FLI1 knockdown. A double-knockdown of EWS-FLI1 and FOXO1 resulted in downregulation of 306 genes (logFC<−0.7, *P*<0.05). The overlap of this gene set with 973 EWS-FLI1-repressed genes contained 94 genes (*P*<10^−10^, hypergeometric test). Upon overexpression of AKT- or CDK2-phosphorylation-resistant FOXO1 mutants, 208 and 184 genes, respectively, were upregulated by FOXO1 (logFC>0.7). Note that the levels of ectopically expressed FOXO1 constructs were similar to endogenous FOXO1 expression after doxycycline-induced EWS-FLI1 knockdown (Figure 4e). Of these, 94 and 58 genes, respectively, were also found upregulated upon EWS-FLI1 knockdown. These overlaps are highly significant (*P*<10^−10^, hypergeometric test). Thus, a significant overlap between FOXO1-activated and EWS-FLI1-repressed genes was identified as measured by three different approaches. Validation of these results was performed by reverse transcription–quantitative PCR on three arbitrarily chosen genes (*EPAS1* and *MME* also show FOXO1-binding sites in the TRANSFAC (transcription factor database) from the overlap of genes that were found significant in all three experimental settings using two ES cell lines. Reactivation of endogenous FOXO1 as a consequence of doxycycline-induced EWS-FLI1 knockdown led to the transcriptional induction of all three tested genes in A673sh cells, which was largely abolished upon RNA interference knockdown of FOXO1 (Figures 4b and d and Supplementary Figure 3a). Likewise, significant re-repression of *EPAS1* and *MME* was observed in TC252 cells after EWS-FLI knockdown, whereas *OSMR* did not respond, a cell line-specific difference in the repressive signature of EWS-FLI1 (Figure 4c and Supplementary Figure 3e). Similar results were obtained using a second shRNA targeting endogenous FOXO1 in A673sh cells (Supplementary Figures 3b–d). In contrast to wild-type FOXO1, the introduction of AKT-phosphorylation-resistant FOXO1 significantly increased the expression of *EPAS1* and *MME* in A673sh and TC252 cells, whereas *OSMR* was only induced in A673sh cells (Figures 4f and g and Supplementary Figures 3f and g). In contrast, despite nuclear localization (Supplementary Figure 2c), CDK2-phosphorylation-resistant FOXO1 failed to reactivate these genes (Figures 4f and g). These results are consistent with nuclear CDK2-phosphorylation-resistant FOXO1 remaining subject to AKT-mediated phosphorylation, which was previously reported to lead to functional dissociation from DNA as a consequence of 14-3-3 binding.^18^ We therefore speculate, that even though the CDK2-phosphorylation-resistant FOXO1 mutant accumulates within the cell nucleus, the ability to induce target gene transcription is limited. Taken together, these data confirm our hypothesis that a repressive sub-signature of EWS-FLI1 regulated genes is due to the suppression of FOXO1.

### Functional restoration of nuclear FOXO1 expression in ES results in impaired proliferation and reduced colony formation capability

**Figure 5:**
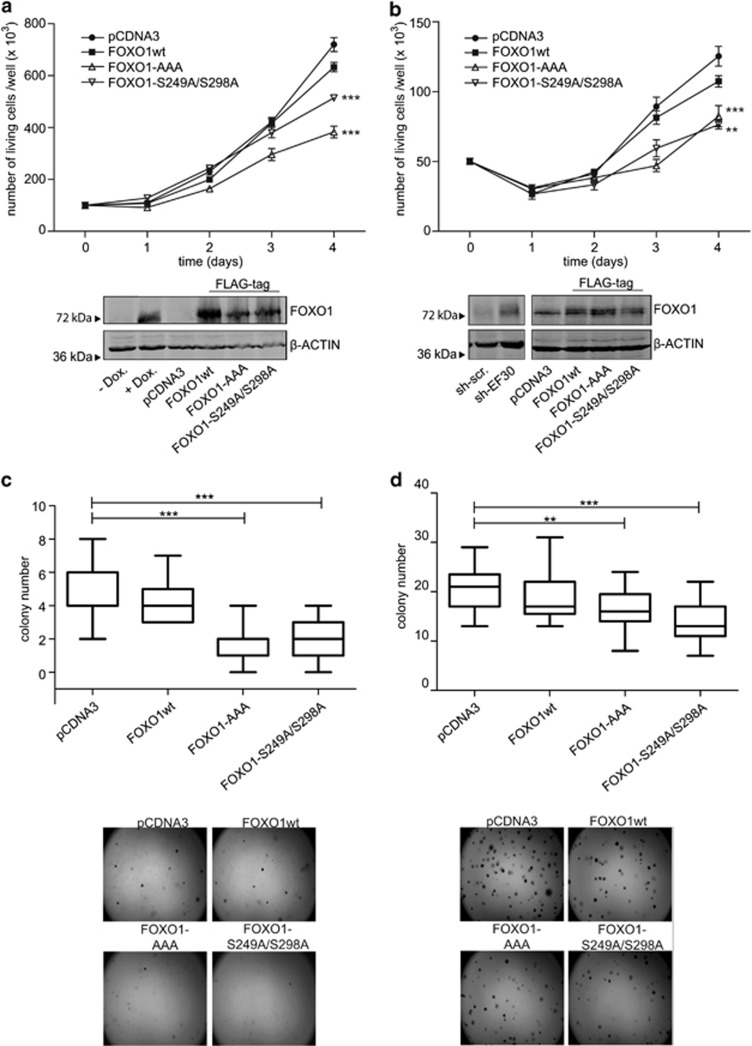
Functional restoration of nuclear FOXO1 expression in ES cells results in impaired proliferation and reduced soft agar colony formation capability. Monolayer growth of (**a**) A673sh, and (**b**) SK-N-MC cells that were transfected with AKT- and CDK2-resistant FOXO1, or the empty vector control or FOXO1wt. Experiments were performed in triplicates and one representative experiment of at least three is shown. Corresponding controls for FOXO1 protein expression are shown below the growth curves. (**c**, **d**) Clonogenicity of A673sh and SK-N-MC cells as studied by soft agar colony formation assays *in vitro*. The corresponding colony sizes were not found to be drastically different between controls (empty vector and FOXO1wt) and nuclear FOXO1 mutants, suggesting significantly reduced clonogenicity triggered by nuclear FOXO1. Colonies were counted 14 days after seeding. **P<0.01 and ***P<0.001.

As FOXO proteins are known to regulate the cell cycle inducing G1 arrest,^19^ we asked whether the restoration of nuclear FOXO1 functionally affects the proliferative activity of ES cells *in vitro*. A673sh and SK-N-MC cells were transfected with CDK2- and AKT-phosphorylation-resistant FOXO1 in the presence of EWS-FLI1. Control cells were transfected with empty vector or FOXO1wt. Proliferation of viable cells was monitored 4 days post transfection. As shown in Figures 5a and b, both A673sh and SK-N-MC exhibited significantly reduced proliferation upon restoration of nuclear FOXO1 compared with cells transfected with the empty vector control or FOXO1wt. The ability of cells to grow under anchorage-independent conditions is considered a hallmark of oncogenic transformation.^20^ When seeded into soft agar, the number of colonies formed by A673sh and SK-N-MC cells after 2 weeks was significantly reduced upon expression of nuclear FOXO1 compared with FOXO1wt and the empty vector control (Figures 5c and d). As colony sizes did not differ dramatically between control-transfectants and cells expressing nuclear-targeted FOXO1, the observed significant reduction in colony number cannot be solely attributed to reduced proliferation but to decreased clonogenicity. Taken together, these results suggest that FOXO1 has an important role in ES oncogenesis.

### Methylseleninic acid reactivates endogenous FOXO1 in a dose- and time-dependent manner

As we found endogenous FOXO1 to be negatively regulated by EWS-FLI1 at transcriptional and post-translational levels in ES, a chemical compound, methylseleninic acid (MSA), previously shown to reactivate FOXO1 in prostate cancer cells,^21^ was investigated in a proof of principle study for its potential to reactivate FOXO1 activity in ES cells.

**Figure 6:**
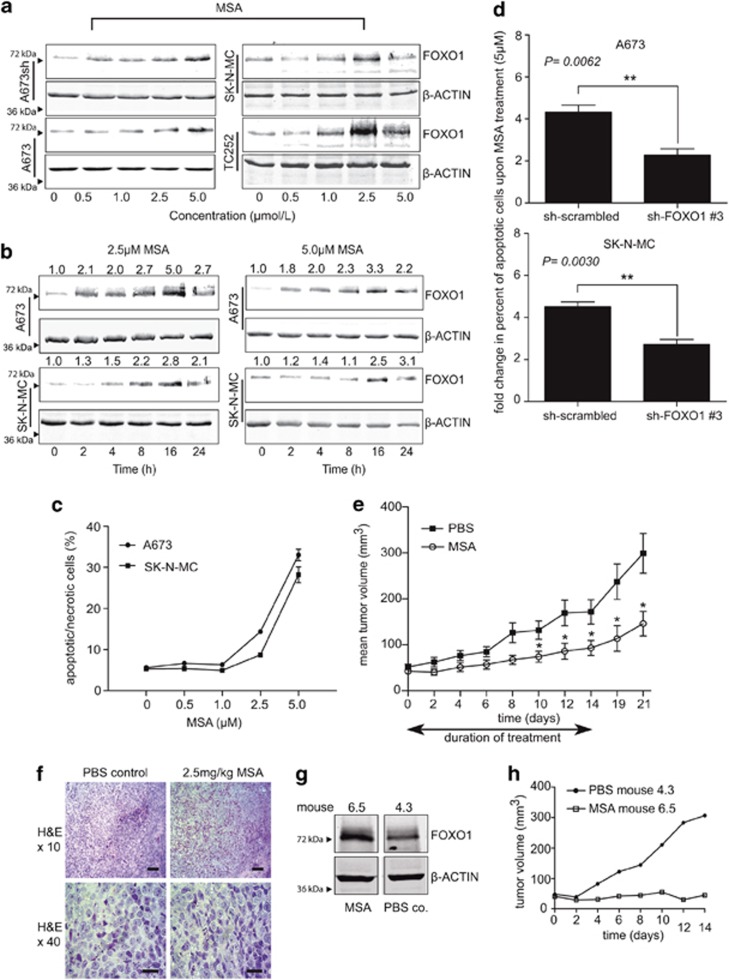
MSA reactivates FOXO1 expression, induces ES cell death *in vitro* and reduces ES tumor growth *in vivo*. MSA dose- (**a**) and time-dependent (**b**) FOXO1 protein induction as monitored by immunoblotting in different ES cell lines as indicated. Protein expression in (**b**) was quantified using the LICOR Odyssey Infrared Imaging System. (**c**) MSA induces dose-dependent cell death *in vitro* as measured by flow cytometry after 16 h of treatment. (**d**) MSA-induced cell death of A673 and SK-N-MC cells can be partially rescued by transient knockdown of endogenous FOXO1. Results represent the mean±s.e.m. of four experiments performed, and statistical analysis was done using the unpaired *t*-test. (**e**) MSA reduces ES tumor growth *in vivo*. After orthotopic injection of 2 × 10^6^ SK-N-MC cells in the m. gastrocnemius mice were either treated with 2.5 mg/kg MSA (*n*=9) or phosphate-buffered saline (PBS) (*n*=12) every second day for 14 days. Tumor growth was further monitored until day 21. A distribution-free test for tumor growth curve analyses revealed an overall significant difference between the MSA group versus the PBS group (*P*=0.0403). Furthermore, significant differences were observed between the MSA group and the PBS group at different times as measured by the Wilcoxon two sample test (day 10, *P*=0.04283; day 12, *P*=0.03604; day 14, *P*=0.04283; day 19, *P*=0.02518; and day 21, *P*=0.01565). *P<0.05 and **P<0.01. (**f**) Representative hematoxylin and eosin (H&E) stains of tumors from mice treated with PBS (mouse 4.3) or with 2.5 mg/kg MSA (mouse 6.5) every second day for 2 weeks. Scale bar: 100 μm (first row); 20 μm (second row). (**g**) Immunoblot analysis on the tumors from (**f**) showed that MSA treatments induced FOXO1 expression compared to the PBS control. (**h**) Tumor volume over time from mice shown in (**f**) and (**g**). Whereas PBS treatments led to exponential tumor growth, MSA treatments prohibited ES tumor growth consistent with induced FOXO1 expression.

Four different ES cell lines were treated with increasing MSA concentrations and the levels of endogenous FOXO1 protein were monitored by immunoblotting. As shown in Figure 6a, FOXO1 was induced in a concentration-dependent manner with highest expression between 2.5 and 5 μM MSA. FOXO1 protein expression peaked at 16 h (Figure 6b), whereas mRNA levels were already induced between 2 and 4 h of MSA treatment and declined gradually thereafter (Supplementary Figures 4a and b). These findings suggest that MSA not only induces FOXO1 protein expression but also regulates FOXO1 at the mRNA level. As shown in Figure 6b, the quantification of FOXO1 protein expression revealed a three to fivefold induction upon MSA treatment which was comparable with FOXO1 protein levels after the EWS-FLI1 knockdown (three to sixfold, data not shown). Notably, MSA treatments led to nuclear FOXO1 expression in the presence of EWS-FLI1 both *in vitro* (Supplementary Figures 4c and d), and *in vivo* (Supplementary Figure 4e). This observation was accompanied by reduced cytoplasmic P-FOXO1 levels, presumably as a consequence of MSA-induced reduction of P-AKT.^22^

### MSA induces cell death which is dependent on FOXO1 expression and reduces tumor growth *in vivo*

To analyze the effect of MSA on ES cell survival, A673 and SK-N-MC cell lines were treated with different concentrations of MSA for 16 h and subsequently processed for flow cytometric cell death analysis. Apoptotic (Annexin-V-positive cells) and DAPI (4',6-diamidino-2-phenylindole)-positive necrotic cells were counted together to monitor overall cell death induction as shown in Figure 6c. At a final concentration of 5 μM MSA, the proportion of drug-induced cell death approached 30–40% in both cell lines.

To clarify whether FOXO1 was involved in MSA-induced cell death, we transiently transfected SK-N-MC and A673 ES cells with either sh-scrambled control or sh-RNA targeting endogenous FOXO1 followed by MSA treatment (5 μM) for 24 h (Supplementary Figures 5a and b). In both cell lines, induction of cell death was induced in sh-scrambled controls, whereas the knockdown of endogenously induced FOXO1 significantly reduced the apoptotic MSA effect, suggesting that FOXO1 is at least partially involved in MSA-induced cell death (Figure 6d).To assess the potential of MSA to reduce ES tumor growth *in vivo*, we used an orthotopic mouse xenotransplantation model. SK-N-MC cells (2 × 10^6^) were directly injected into the m. gastrocnemius of severe combined immune deficiency/bg mice. Mice bearing tumors bigger than 100 mm^3^ at day 0 were excluded from the analysis to avoid heterogeneity in MSA take-rate.^23^ Mice were observed for a period of 21 days after starting MSA treatments. As shown in Figure 6e, tumor growth was significantly reduced in the MSA group compared with phosphate-buffered saline control mice. The tumors exhibited a small round cell phenotype characteristic of ES, as shown by representative hematoxylin and eosin stains (Figure 6f).

Notably, the MSA-mediated inhibitory growth effect on ES tumors persisted beyond the treatment period and tumor growth delay was accompanied by increased FOXO1 protein levels (Figures 6g and h), suggesting a potent anti-tumorigenic activity of MSA.

## Discussion

EWS-FLI1 has been proposed as the ideal therapeutic target in ES,^24^ but hitting nuclear transcription factors by small molecules remains a challenge. Intervention with the downstream transcriptional network of EWS-FLI1 may offer an alternative treatment option. Very little is known about the mechanisms of EWS-FLI1-mediated gene repression, even though there is evidence that part of its repressive signature can be assigned to activation of transcriptional repressors NKX2.2 and NR0B1,^25, 26^ to epigenetic mechanisms involving the NuRD (Nucleosome remodelling and deacetylation) complex^27^ or to post-transcriptional mechanisms.^28^

**Figure 7:**
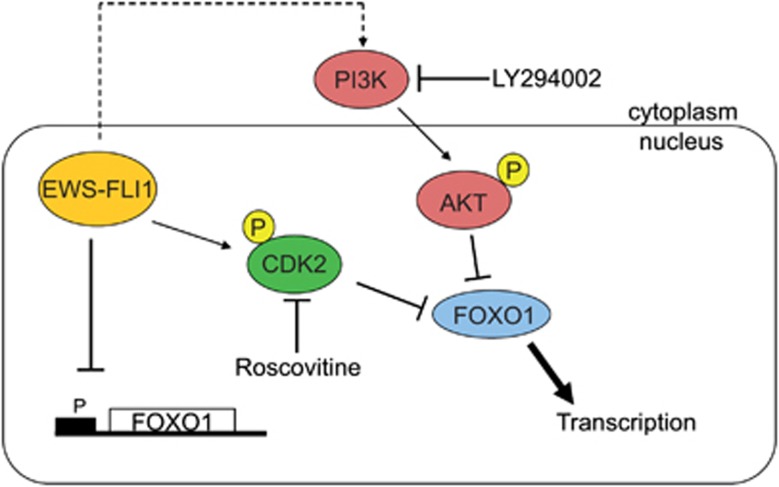
The transcriptional activity of FOXO1 is regulated in a multilayered manner. EWS-FLI1 not only represses the transcription of FOXO1 but also affects P-AKT levels and regulates CDK2 activity, thus regulating FOXO1 transcriptional activity on a post-translational level. Blocking the PI3K-AKT signaling pathway with the canonical PI3K inhibitor LY294002 or by blocking active CDK2 with roscovitine can overcome the repressive effect of EWS-FLI1 as shown for ectopically expressed FOXO1wt.

Here, we report that EWS-FLI1 exerts a significant part of its transcriptional repression activity via inhibition of the forkhead transcription factor FOXO1. Our *in vitro* data show that FOXO1 is tightly regulated in a multilayered manner by EWS-FLI1-mediated transcriptional repression and inhibitory phosphorylation by EWS-FLI1-induced CDK2 and AKT (summarized in Figure 7).

There is ample experimental evidence that AKT and CDK2 regulate the subcellular localization of FOXO1 independently of each other. In ES, the PI3K pathway is essential for cellular survival,^29, 30^ activated by growth factors such as insulin-like growth factor-1. It was previously shown that insulin-like growth factor-binding protein is directly suppressed by EWS-FLI1 sustaining the activation of this signaling pathway.^31^ Treatment with recombinant insulin-like growth factor-binding protein-3 drastically decreased levels of phosphorylated AKT in ES cells^31^ as a consequence of decreased phosphorylation of insulin-like growth factor-1 receptor.^32^ Consistent with these findings, our analysis revealed that using a phosphorylation-resistant version of FOXO1, AKT-mediated nuclear exclusion was abrogated. On the other hand, CDK2 was reported to functionally interact with FOXO1 leading to phosphorylation at two specific serine residues (S249/S298) followed by nuclear exclusion.^16^ Interestingly, chromatin immunoprecipitation-seq (not shown), expression and reporter-gene analyses (Supplementary Figure 1c) identified CDK2 as an EWS-FLI1-induced target, suggesting an important role for CDK2 in FOXO1 repression by EWS-FLI1. Here, we show that knockdown of CDK2 was sufficient to drive ectopically expressed FOXO1 into the nucleus of ES cells even in the presence of active PI3K/AKT signaling. This finding is supported by nuclear localization of a CDK2-resistant FOXO1 mutant (S249A/S298A). AKT phosphorylation at residues T24 and S256 causes the binding of chaperon protein 14-3-3,^11^ potentially masking the nuclear localization signal and causing dissociation of FOXO1 from DNA.^18^ In contrast, CDK2 phosphorylation at S249 does not affect 14-3-3 binding^16^ but increases the negative charge of an adjacent stretch of three arginines within the nuclear localization signal,^16^ which was previously demonstrated to have a major role in FOXO3 nuclear localization.^33^ However, CDK2-phosphorylation-resistant FOXO1 is still exposed to AKT-mediated phosphorylation thus allowing for 14-3-3-mediated destabilization of FOXO1 on DNA. Therefore, it was not unexpected that overexpression of CDK2-mutant FOXO1 did not reactivate EWS-FLI1-repressed genes to the same extent as AKT-phosphorylation-resistant FOXO1. The latter was capable of reactivating about 10% of EWS-FLI1-repressed genes as opposed to only 6% reactivated by CDK2-resistant FOXO1. FOXO1 suppression by RNA interference prohibited the activation of a similar number of genes by EWS-FLI1 knockdown as by the AKT resistant mutant. Still, EWS-FLI1 knockdown was more efficient in reactivating FOXO1 target genes than individual phosphorylation-resistant mutants. This difference is likely due to a so far unrecognized modulating effect of the multiple serine-to-alanine amino-acid substitutions on the transcriptional activity of the mutant proteins. FOXO1 was demonstrated to be involved in cellular differentiation (reviewed in Accili and Arden^34^), and more specifically to function as an early molecular regulator in the differentiation of mesenchymal cells into osteoblasts.^35^ Along these lines, long-term inhibition of EWS-FLI1 enables the differentiation of ES cells into the osteogenic lineage when treated with a suitable differentiation cocktail.^5^ Thus, reactivation of FOXO1 may represent an appropriate tool to induce differentiation in ES. Consistent with FOXO1-driven regulation of proliferation via induction of cell-cycle arrest,^36^ our *in vitro* assays revealed reduced proliferative and clonogenic ability of ES cells upon restoration of nuclear FOXO1, suggesting that FOXO1 suppression has an important role in ES oncogenesis.

We therefore hypothesized that reactivation of endogenous FOXO1 in the presence of EWS-FLI1 may constitute a potentially promising therapeutic strategy for ES. As a proof of principle, we chose to interrogate a small molecule, MSA, for its activity in ES cells *in vitro* and *in vivo*.

We found that ES cell lines were highly sensitive to MSA treatment. MSA was previously shown to be involved in the induction of apoptosis by different mechanisms.^37, 38^ We here demonstrate that cell death induction in ES was at least partially dependent on MSA-induced FOXO1 activity. We also observed antitumor activity in an orthotopic ES xenograft model which was accompanied by elevated FOXO1 protein levels consistent with our hypothesis that FOXO1 activation downstream of EWS-FLI1 confers a therapeutic benefit. However, the mechanism of FOXO1 reactivation by MSA remains elusive. MSA was demonstrated to reduce insulin-like growth factor-1 receptor levels and, consequently, phospho-AKT levels in a mouse mammary hyperplastic epithelial cell-line^39^ providing a possible explanation for the post-translational FOXO1-inducing activity of the drug. A study in prostate cancer cells revealed that MSA treatment results in decreased expression of genes involved in metabolism, angiogenesis, certain transcription factors and, interestingly, signal transduction (ERK and AKT), which was significantly higher in tumor cells than in non-tumor cells.^40^

High selenium doses are often associated with intoxication, and the MSA concentration used in this study (2.5 mg/kg) represents the highest tolerated dose in severe combined immune deficiency/bg mice (data not shown). For potential clinical use, we envision the combination of low-dose methylated selenium in combination with other standard chemotherapeutic agents, as it was reported that MSA synergizes with conventional drugs such as etoposide and doxorubicin, which are frequently used in the treatment of ES.^40^

Taken together, our results imply that FOXO1 acts as a tumor suppressor in ES, and identify FOXO1 reactivation as a promising strategy for a future ES-specific therapy.

## Materials and methods

### Cell culture

ES cell lines SK-N-MC, TC252, A673 and its doxycycline-inducible EWS-FLI1-shRNA expressing derivative A673sh were previously described.^21, 41^ Cells were authenticated by PCR. The CDK2 inhibitor roscovitine, methylseleninic acid (Sigma-Aldrich, St Louis, MO, USA) and the PI3K/AKT inhibitor LY294002 (Merck, Darmstadt, Germany) were used at concentrations and for time periods indicated in the figures.

### Plasmids and transfection

The pCDNA3-FLAG-FOXO1wt plasmid was a gift from Dr Guan (Department of Pharmacology, University of California, San Diego, CA, USA). The plasmid encoding CDK2 phosphorylation-resistant Flag-FOXO1 mutant (pCDNA3.1-FLAG-FOXO1-S249A/S298A) was obtained from H Huang and DJ Tindall (Department of Biochemistry and Molecular Biology, Rochester, MI, USA). pCDNA3-FLAG-FOXO1-AAA (T24A/S256A/S319A)^17^ and pECE-FLAG-FOXO3 wt^42^ constructs were purchased from Addgene (Cambridge, MA, USA). shRNA to CDK2 (PBS/U6shCDK2) and empty vector control (pBS/U6 empty) constructs were provided by Dr Shi (Harvard Medical School, Boston, MA, USA). FOXO1-targeting shRNAs (no. 1, 5′-TGACTTGGATGGCATGTTCATTGAGCGCT-3′ no. 3, 5′-GAAGAGCTGCATCCATGGACAACAACAGT-3′) and sh-scrambled control plasmids were from OriGene Technologies (Rockville, MD, USA). Mouse stem cell virus-based retroviral construct mouse stem cell virus-MIGR1 expressing low levels of enhanced green fluorescent protein^32^ was provided by M Busslinger (Institute of Molecular Pathology, Vienna, Austria). The plasmid encoding shRNA to EWS-FLI1 (shEF30) has previously been described.^43^

Cells were transfected using the Lipofectamine Plus reagent (Invitrogen, Groningen, the Netherlands).

### Immunofluorescence analysis

ES cells were processed for immunofluorescence analysis following standard fixation (4% paraformaldehyde) and washing steps and subsequently stained with anti-FLAG antibody (DYKDDDDK, Cell Signaling Technology Inc., Danvers, MA, USA) and a secondary red-fluorescent Alexa Fluor 594 goat anti-rabbit IgG antibody (Molecular Probes, Eugene, OR, USA).

### RNA preparation and reverse transcription–quantitative PCR

Total RNA was prepared with a Qiagen RNAeasy kit (Qiagen, Hilden, Germany). Transcripts were quantified by TaqMan reverse transcription–quantitative PCR using the ABI Prism 7900 Detection System (Applied Biosystems, Foster City, CA, USA). Primers and probes used for reverse transcription–quantitative PCR are listed in Supplementary Table S1.

### Immunoblot analysis

Total proteins (30–50 μg) were resolved by 8.5% SDS-polyacrylamide gel electrophoresis and processed for immunoblotting. The following antibodies were used: FOXO3 (H-144) and CDK2 (sc-163) (Santa Cruz, CA, USA), FOXO1 (C29H4), Phospho-FoxO1-Ser256 (9461), Phospho-FOXO3-Ser253 (9466), Lamin A/C (2032), AKT (9272) and p-AKT-Thr308 (244F9) antibodies from Cell Signaling Technology (New England Biolabs GmbH, Frankfurt, Germany). Antibodies to β-actin and α-Tubulin (DM1A) were from Abcam (Cambridge, UK) and Calbiochem (Merck, Darmstadt, Germany), respectively. FLI1 antibody was from MyBioSource (San Diego, CA, USA). For preparing fractionated cell extracts, the Nuclei EZ prep nuclei isolation kit (NUC-101, Sigma, St Louis, MO, USA) and the NE-PER Nuclear and Cytoplasmic Extraction Reagents (Pierce Biotechnology, Rockford, IL, USA) for fresh tumor tissue were used. Linear protein quantification was performed using the LICOR Odyssey Infrared Imaging System.

### Microarray and *in-silico* motif analysis

Gene-expression profiling was performed using Affymetrix HG-U133-PLUS2 arrays (Affymetrix Inc., Santa Clara, CA, USA). Microarray data were obtained in compliance to Minimum information about a microarray experiment (MIAME) guidelines and submitted to Gene Expression Omnibus accession number GSE37409. All further analyses were performed in R statistical environment using Bioconductor packages.^44^ Affymetrix CEL files were preprocessed as described previously,^6^ yielding a final number of 8154 probe sets that were used for all further analyses. Differentially expressed genes were determined using a moderated *t*-test in the R package ‘limma'.^45^ All *P*-values were corrected for multiple testing using the ‘Benjamini-Hochberg' correction method.

Time-course analysis of gene expression in A673sh was performed on Affymetrix HGU-133A2 arrays at five time points: 0, 18, 36, 53 and 72 h. Each experiment was reiterated at least twice and the EWS-FLI1 protein was found to be consistently downregulated already at 18 h in all replicate experiments. After filtering out probe sets with very low expression values across all samples (R package ‘panp'), for every gene the most informative probe set was selected (R package ‘genefilter') yielding 8023 genes for further analysis. Differential expression for each gene at each later time point against 0 h was determined using a moderated *t*-test from the R package ‘limma'. The resulting *P*-values were corrected for multiple testing using the Benjamini-Hochberg method.

### Promoter analysis for binding sites of known transcription factors

Coordinates of all conserved transcription factor binding sites were downloaded from the UCSC (University of California, Santa Cruz) genome browser database (tfbsConsSites.txt) and hits for all sites within 1 kb upstream of the transcription start site of all genes in the refGene.txt table were counted (both tables were downloaded from: http://hgdownload.cse.ucsc.edu/goldenPath/hg18/database/). Subsequently, the occurrence of each transcription factor binding sites was correlated with gene expression change over time (all later time points vs 0 h across all genes after the EWS-FLI1 knockdown). To count motifs in genes with similar time-course dynamics, genes were ordered by their logFC and binned into 100 equally sized bins (R package ‘dr'). Within these bins, motifs of all transcription factor binding sites were counted and plotted against the average logFC of the respective bin. Pearson correlation coefficients between number of motifs and mean logFC were recorded.

### Proliferation assay

ES cells were seeded in triplicates at 5 × 10^4^ cells/35-mm dish and proliferation of viable cells was monitored by cell counting using a Bürker-Türk chamber in combination with trypan-blue exclusion test for 4 days.

### Soft agar assay

Experiments were conducted and analyzed as described previously.^46^

### Flow cytometric analysis

For testing ES sensitivity to different concentrations of MSA a no-wash sample preparation was used. Each cell suspension was stained for Annexin-V using FITC Annexin-V antibody (BD Biosciences, Schwechat, Austria). After staining, cells were acquired on a BD LSRII flow cytometer.

For cell death rescue experiments using sh-FOXO1 and sh-scrambled control, cells were co-transfected using the low-expression enhanced green fluorescent protein-Plasmid (mouse stem cell virus-MIGR1). Forty-eight hours post transfection, cells were treated or not with 5 μM MSA for 24 h and subjected to flow cytometric cell-viability analysis. For data evaluation the FlowJo software (Version 7.6.3; Tree Star Inc., Ashland, OR, USA) was used.

### Chromatin immunoprecipitation assay

Chromatin immunoprecipitation experiments were carried out using MAGnifyTM Chromatin Immunoprecipitation System Kit (Invitrogen). For immunoprecipitation, 15 μl of FLI1 antibody (MyBioSource, San Diego, CA, USA) were used. PCR was performed using Phusion Hotstart II polymerase (Finnzymes, Espoo, Finland). Primers used in 40 cycles of amplification are listed in Supplementary Table S2.

### *In vivo* studies

An orthotopic xenotransplantation model using the ES cell-line SK-N-MC was used as described in.^47^ Tumor formation was examined on a daily basis and MSA treatments begun when tumors reached measurable sizes. Two groups, consisting of 14 mice each, were either intraperitoneally injected with 2.5 mg/kg MSA or phosphate-buffered saline and treatments were conducted every second day for 2 weeks. Experiments were terminated when tumors reached the critical volume of 1500 mm^3^. Mice studies were approved by the state regulatory board and all animals received humane care in compliance with the respective guidelines.

### Statistical analysis of *in vitro* and *in vivo* assays

*In vitro* data were analyzed by the unpaired *t*-test with Welch's correction or with the one-sample *t*-test using the Prism 5 for Windows (version 5.02) statistical software (GraphPad Prism Software Inc.). Data shown in graphical format represent the means (±s.e.m.), and a *P*-value of <0.05 is considered statistically significant.

To test for overall differences in the growth curves of MSA treated vs untreated mice, a distribution-free permutation test was utilized as described.^48^ In addition, a mixed linear model analysis was performed on log-transformed values. To test for differences between treated and untreated groups on each day separately, a Wilcoxon signed rank test was performed. The mixed linear model analysis and Wilcoxon tests were done in SPPS.
