# Supplementary figures and images for: Suppression of FOXO1 is responsible for a growth regulatory repressive transcriptional sub-signature of EWS-FLI1 in Ewing sarcoma
Source: Oncogene. 2013 Sep 2;33(30):3927–38. doi: 10.1038/onc.2013.361 (PMC4114138; doi:10.1038/onc.2013.361)

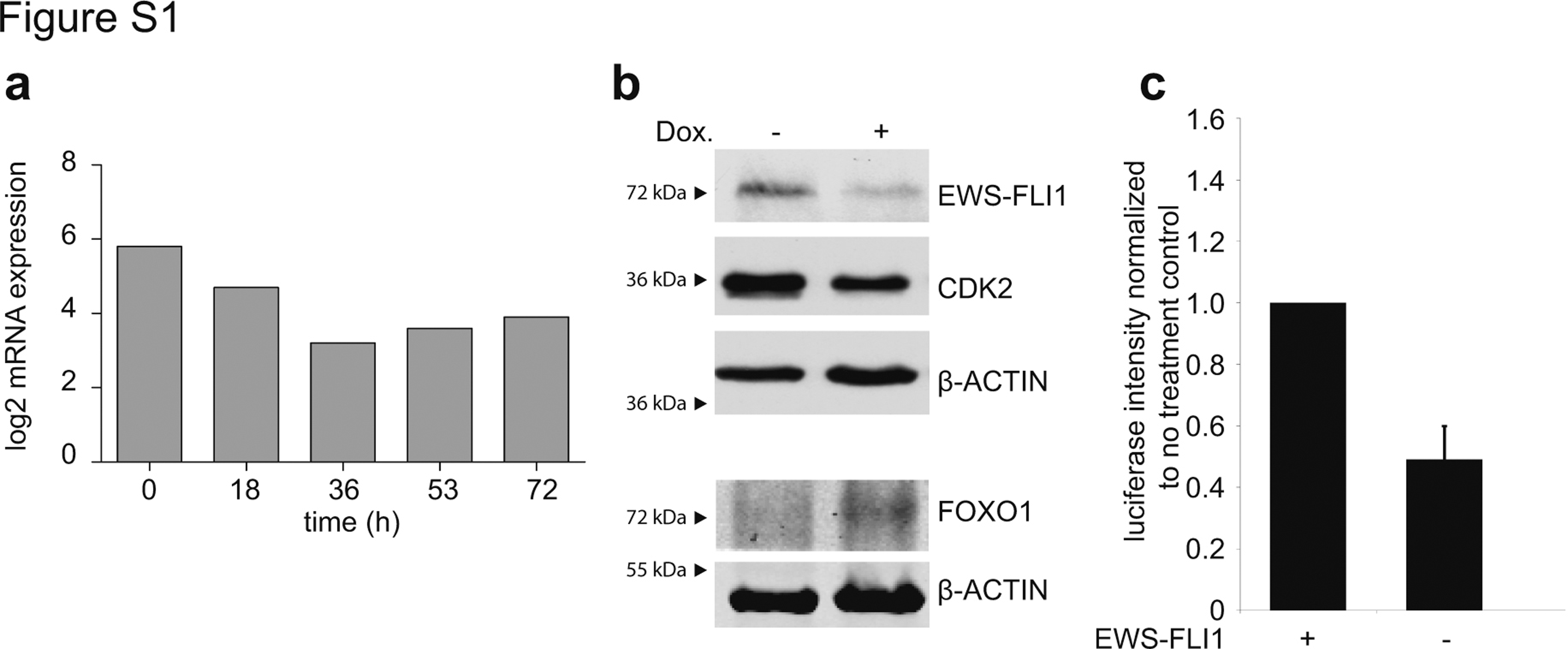

Supplement: Supplementary Figure S1 [file onc2013361x1.tif]

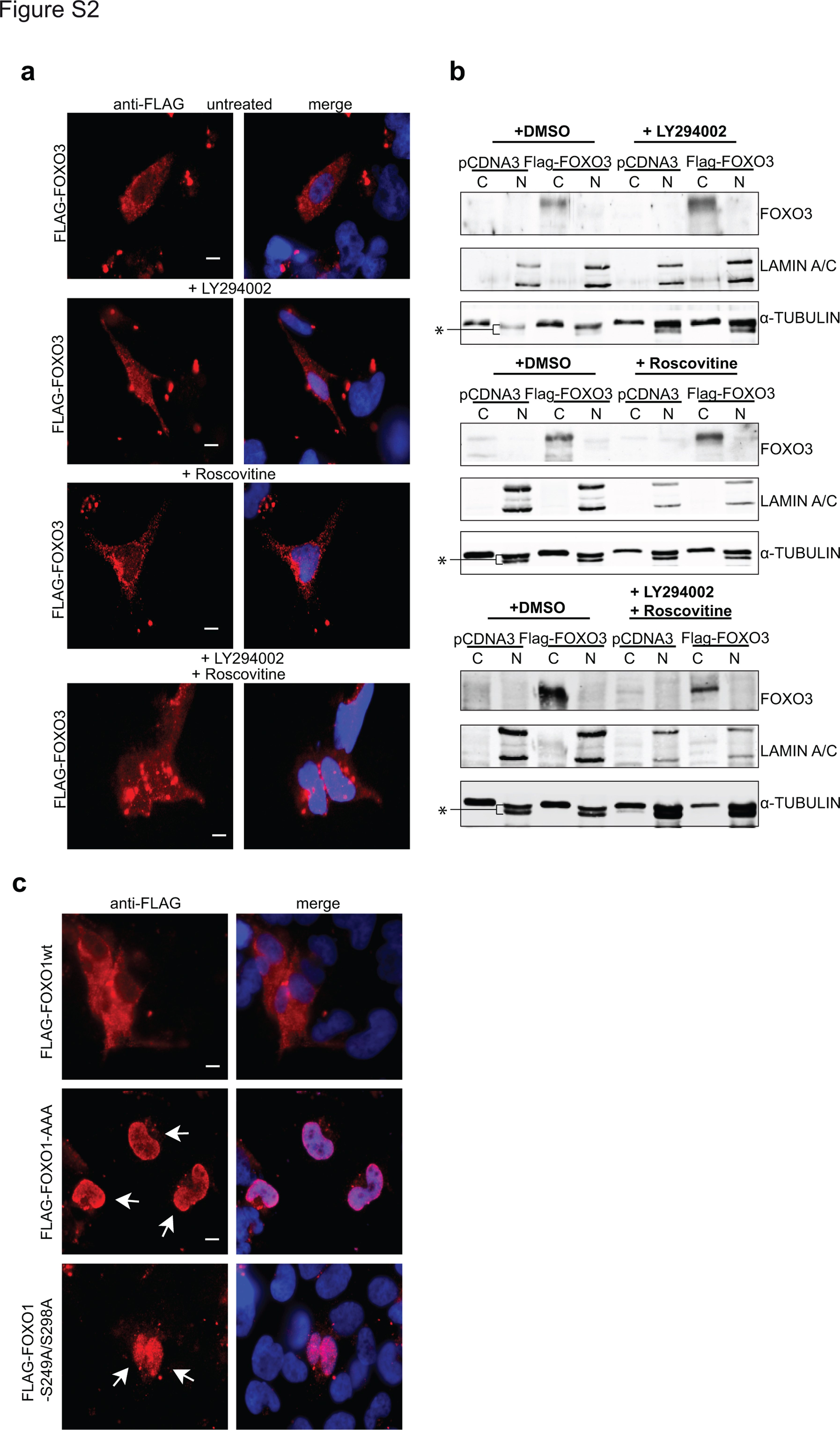

Supplement: Supplementary Figure S2 [file onc2013361x2.tif]

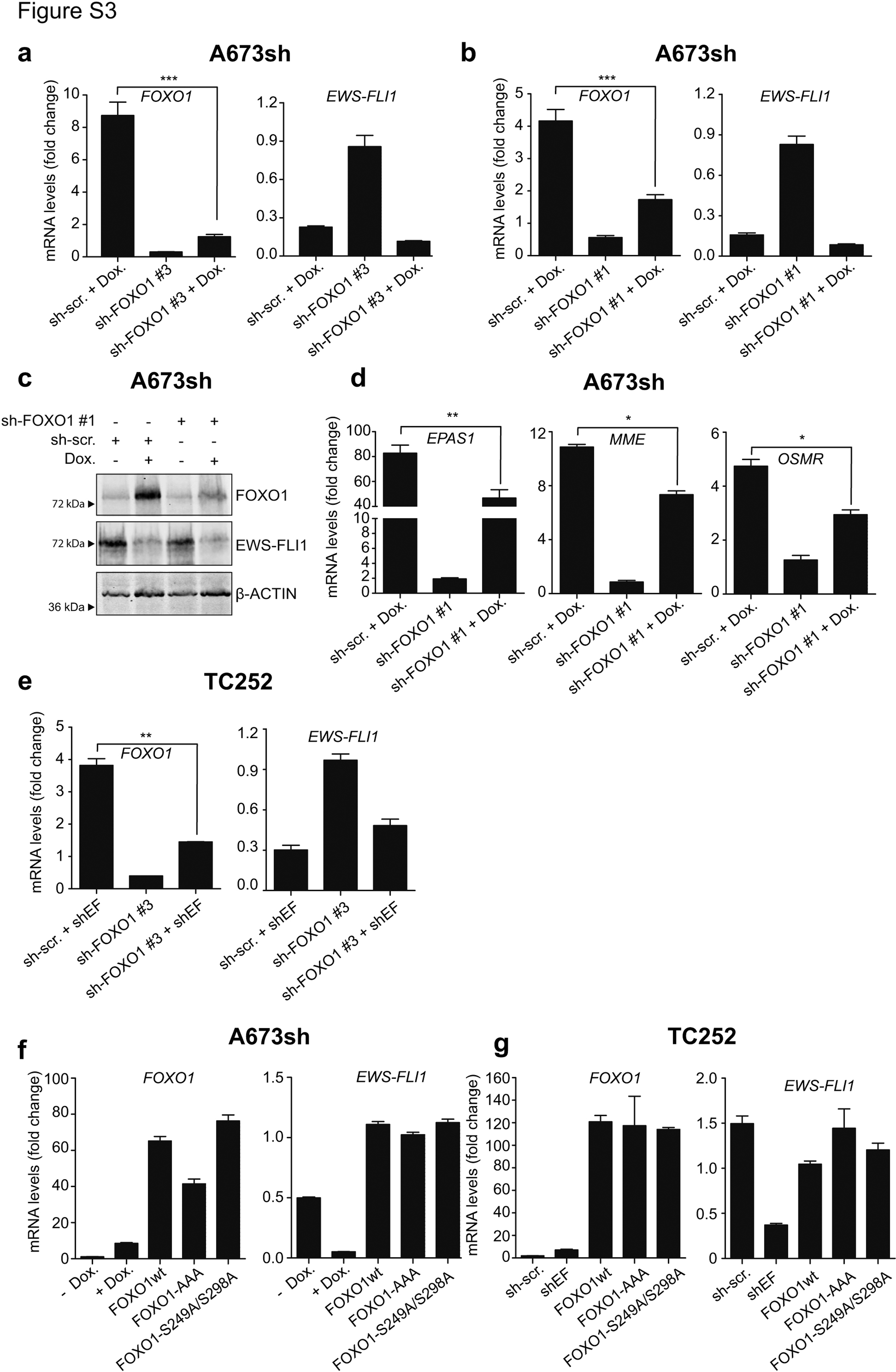

Supplement: Supplementary Figure S3 [file onc2013361x3.tif]

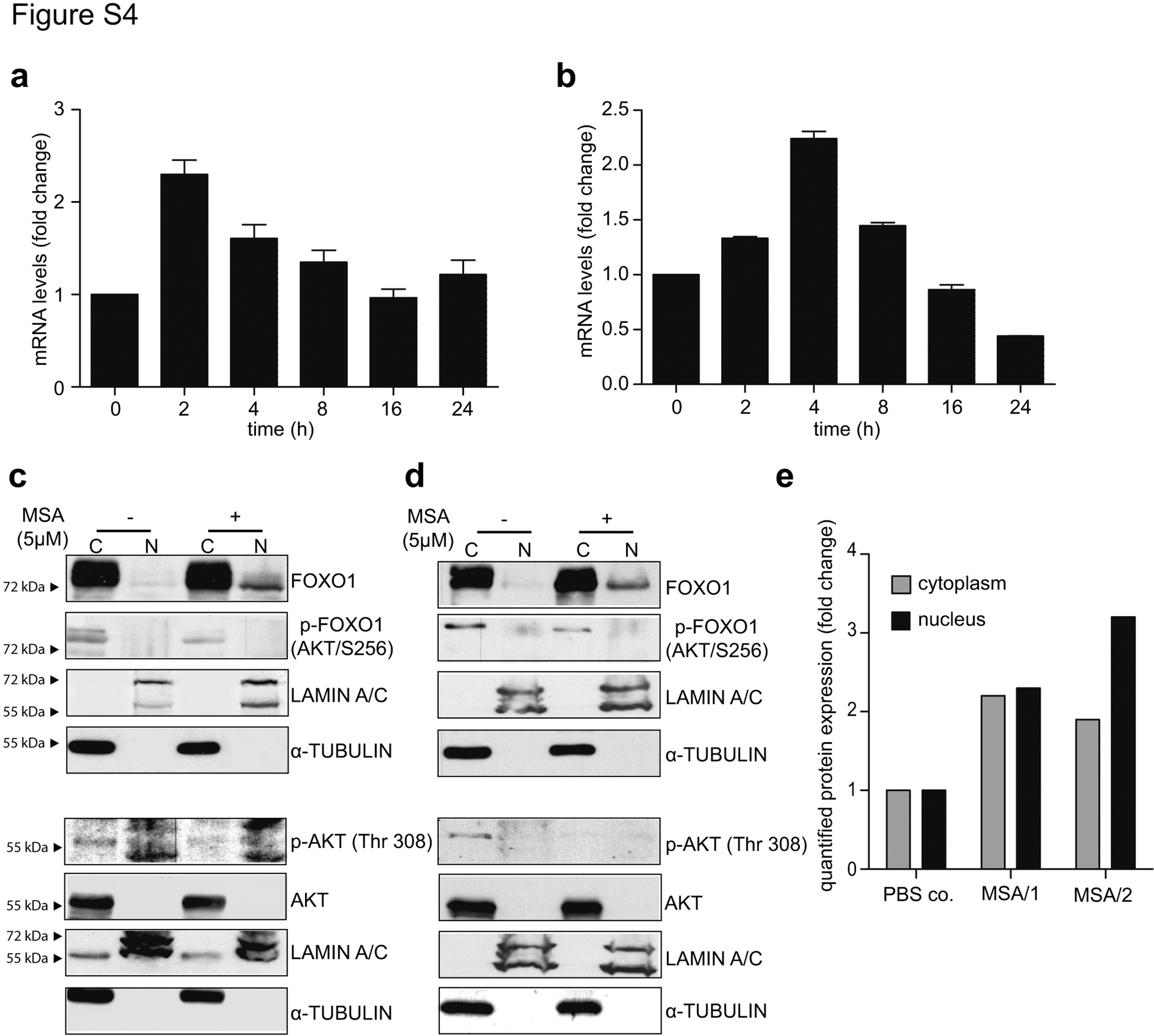

Supplement: Supplementary Figure S4 [file onc2013361x4.tif]

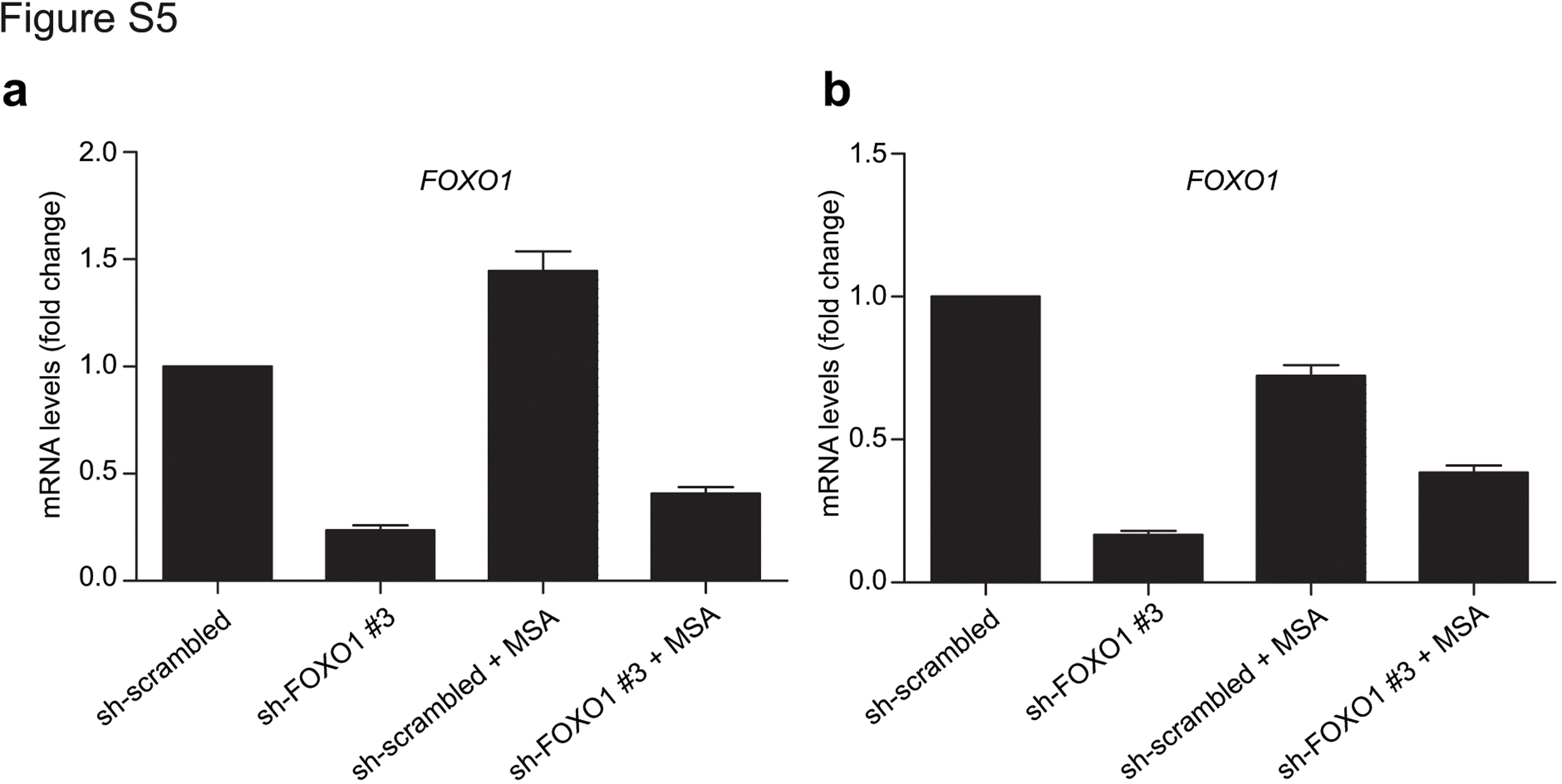

Supplement: Supplementary Figure S5 [file onc2013361x5.tif]
