# Supplementary material for: Suppression of FOXO1 is responsible for a growth regulatory repressive transcriptional sub-signature of EWS-FLI1 in Ewing sarcoma
Source: Oncogene. 2013 Sep 2;33(30):3927–38. doi: 10.1038/onc.2013.361 (PMC4114138; doi:10.1038/onc.2013.361)
Supplement: Supplementary Information [file onc2013361x6.doc]

**Supplementary Data**

**Table S1**

| **gene** | **fw primer** | **rev primer** | |
| --- | --- | --- | --- |
| ***FOXO1*** | AAGAGCGTGCCCTACTTCAA | GTTGTTGTCCATGGATGCAG | |
| ***EPAS1*** | AAGCCTTGGAGGGTTTCATT | TCATGAAGAAGTCCCGCTCT | |
| ***MME*** | CCTTCTTTAGTGCCCAGCAG | CCAGTCAACGAGGTCTCCAT | |
| ***OSMR*** | GTCATCTGGGTGGGGAATTA | CAAAGTGTGTGGCACATTCC | |
| ***EWS-FLI1 (I)*** | CAGCCAAGCTCCAAGTCAATATAG | GCTCCTCTTCTGACTGAGTCATAAGA | |
| ***b2M*** | TGAGTATGCCTGCCGTGTGA | TGATGCTGCTTACATGTCTCGAT | |
| **gene** | **probe** | |  |
| ***FOXO1*** | CGGCGGGCTGGAAGAATTCA | |  |
| ***EPAS1*** | TGACCCAAGATGGCGACATG | |  |
| ***MME*** | CGGCATGGTCATAGGACACG | |  |
| ***OSMR*** | TTCTGCATTGGAGCTGGGAA | |  |
| ***EWS-FLI1 (I)*** | CTGCCCGTAGCTGCTGCTCTGTTG | |  |
| ***b2M*** | CCATGTGACTTTGTCACAGCCCAAGATAGTT | |  |

**Table S2**

| **promoter region** | **fw primer** | **rev primer** |
| --- | --- | --- |
| ***FOXO1* -609/-412** upstream of TSS | GCCCGACTTACGGGATCT | GAGAAAAACACCCCACTACCC |
| ***FOXO1* -961/-736** upstream of TSS | CCGGCGACACTTTGTTTACT | CGTTCAGCAAAGACATCGTG |
| ***FOXO1* -9071/-8888** upstream of TSS | CAGAGTCCCTCGGTCATCTC | TGCGTTGTTGATTTTCTGCT |

**Figure S1. CDK2 is a EWS-FLI1 induced target.**

**(a)** Kinetics of CDK2 mRNA expression in the EWS-FLI1 knockdown time-course of A673sh cells. X-Axis: interval after doxycycline induced EWS-FLI1 knockdown. Y-Axis: log2 CDK2 mRNA expression. (**b**) CDK2 and FOXO1 protein expression in A673sh cells upon 48 hours EWS-FLI1 knockdown. (**c**) Luciferase reporter gene assay testing CDK2 promoter responsiveness to EWS-FLI1 knockdown. A CDK2 (-122/+458 from the TSS) promoter fragment was cloned into the pGL4.10 vector (Promega, Madison, WI). Cells were treated with doxycycline 48h posttransfection, and RGA were carried out using Bright Glo Luciferase assay kit (Promega, Madison, WI) 96h after transfection (48h after doxycycline induction). Y-axis: promoter activity of doxycycline treated relative to untreated cells (normalized for transfection efficiency and empty vector control transfections). Means and standard deviations of at least three independent experiments, each performed in triplicate, are shown.

**Figure S2. FOXO3 is not subject of EWS-FLI1 mediated gene repression but**

**nuclear localization of FOXO1 is rescued by mutation of inhibitory CDK2 and P-AKT phosphorylation sites.**

(**a**) Representative immunofluorescence analysis of A673sh cells transfected with Flag-FOXO3 and treated with 40µM of the PI3K/AKT inhibitor LY294002 or 40µM of the canonical CDK2 inhibitor roscovitine, or a combination of both 48h post-transfection for 24h. (**b**) A673sh cells were transfected with Flag-FOXO3 as described in (**a**) and cytoplasmic/nuclear fractionations were analyzed by immunoblotting. Notably, FOXO3 did not translocate into the nucleus as shown by two different methods. (*) Indicates unspecific bands in nuclear fractions when probed with anti-tubulin antibody. Note, these bands migrate at slightly different size than cytoplasmic tubulin. (**c**) A673sh cells were transfected with Flag-FOXO1wt, an AKT-resistant (T24A/S256A/S319A, (AAA)) or a CDK2-resistant version of Flag-FOXO1 (S249A,S298A), and immunofluorescence analysis was performed 48h post-transfection. Cells were stained by anti-Flag antibody and visualized by red-fluorescent Alexa Fluor 594 goat anti–rabbit IgG antibody.

Scale bars: 10µM

**Figure S3. mRNA expression of endogenous FOXO1 and of EWS-FLI1 upon modulation of EWS-FLI1 and FOXO1 in A673sh and TC252 cells (related to Figure 4b,c and f,g).**

(**a-b**) Doxycycline induced knockdown of EWS-FLI1 with concomitant FOXO1 silencing using two different FOXO1-shRNAs (#1, #3). (**c**) Representative FOXO1 protein expression showing specificity and efficiency of sh-RNA #1 mediated FOXO1 silencing and (**d**) comparable mRNA expression of the same genes (shown in Figure 4b-c) upon knockdown of FOXO1 using shRNA #1. (**e**) sh-RNA targeting EWS-FLI1 (sh-EF) and concomitant FOXO1 silencing using FOXO1-shRNA#3 in TC252 cells. Statistical relevance was analyzed using the unpaired t-test, B2M was used as internal control and the mRNA expression was normalized to sh-scrambled control.

(**f-g**) FOXO1 and EWS-FLI1 mRNA expression upon ectopic expression of wild-type and mutant nuclear FOXO1 in A673sh and TC252 cells. The mRNA expression was normalized to pCDNA3 empty vector control, B2M was used as internal control and doxycycline or shEF was applied for 72h. Results represent the mean ± SEM of two experiments performed. *p<0.05, **p<0.01 and ***p<0.001.

**Figure S4. FOXO1 mRNA expression is induced early upon treatment with MSA which induces expression of nuclear FOXO1 in the presence of EWS-FLI1 *in- vitro* and *in-vivo*.**

Change of FOXO1 mRNA levels in (**a**) SK-N-MC and (**b**) A673 cells as a function of time measured by RT-qPCR. Shown are representative experiments of at least three, and the fold-induction was calculated in comparison to the untreated control and normalized to B2M. The ES cell lines (**c**) SK-N-MC and (**d**) A673 were treated with 5µM MSA for 16h and subsequently processed to extract cytoplasmic and nuclear protein fractions. As shown in (**e**), nuclear FOXO1 expression was also elevated in the tumors of MSA treated (three times in 48h intervals) mice (MSA/mouse-1, MSA/mouse-2) compared to a PBS control. Protein expression was quantified using the LICOR Odyssey® Infrared Imaging System that allows linear quantification.

The protein samples were probed with FOXO1 specific, p-FOXO1 (AKT-S256) and P-AKT (Thr 308) antibodies. LaminA/C and α-Tubulin were used as nuclear and cytoplasmic loading controls, respectively.

**Figure S5.** **Validation of sh-RNA mediated FOXO1 knockdown and effect of MSA on FOXO1 expression (related to Figure 6D).**

FOXO1 mRNA was readily reduced when ES cell lines (**a**) SK-N-MC and (**b**) A673 were transfected with sh-RNA targeting endogenous FOXO1 compared to the sh-scrambled control, as assessed by RT-qPCR. Results are representative of four experiments and the fold induction was calculated based on the untreated control, and B2M was used as internal control.
