# Supplementary material for: Suppression of FOXO1 is responsible for a growth regulatory repressive transcriptional sub-signature of EWS-FLI1 in Ewing sarcoma
Source: Oncogene. 2013 Sep 2;33(30):3927–38. doi: 10.1038/onc.2013.361 (PMC4114138; doi:10.1038/onc.2013.361)
Supplement: Supplementary Information [file onc2013361x7.doc]

**Supplementary Data**

**Table S1. List of primers and probes used for RT-qPCR.**

| **gene** | **fw primer** | **rev primer** | |
| --- | --- | --- | --- |
| ***FOXO1*** | AAGAGCGTGCCCTACTTCAA | GTTGTTGTCCATGGATGCAG | |
| ***EPAS1*** | AAGCCTTGGAGGGTTTCATT | TCATGAAGAAGTCCCGCTCT | |
| ***MME*** | CCTTCTTTAGTGCCCAGCAG | CCAGTCAACGAGGTCTCCAT | |
| ***OSMR*** | GTCATCTGGGTGGGGAATTA | CAAAGTGTGTGGCACATTCC | |
| ***EWS-FLI1 (I)*** | CAGCCAAGCTCCAAGTCAATATAG | GCTCCTCTTCTGACTGAGTCATAAGA | |
| ***b2M*** | TGAGTATGCCTGCCGTGTGA | TGATGCTGCTTACATGTCTCGAT | |
| **gene** | **probe** | |  |
| ***FOXO1*** | CGGCGGGCTGGAAGAATTCA | |  |
| ***EPAS1*** | TGACCCAAGATGGCGACATG | |  |
| ***MME*** | CGGCATGGTCATAGGACACG | |  |
| ***OSMR*** | TTCTGCATTGGAGCTGGGAA | |  |
| ***EWS-FLI1 (I)*** | CTGCCCGTAGCTGCTGCTCTGTTG | |  |
| ***b2M*** | CCATGTGACTTTGTCACAGCCCAAGATAGTT | |  |

**Table S2. Primer list for ChIP-PCR.**

Primers used for covering EWS-FLI1 binding sites within the FOXO1 promoter at position -609/-412 and -961/-736, or, for negative control, a region upstream of the transcription start site (TSS) at position -9071/-8888 not containing ETS binding sites.

| **promoter region** | **fw primer** | **rev primer** |
| --- | --- | --- |
| ***FOXO1* -609/-412** upstream of TSS | GCCCGACTTACGGGATCT | GAGAAAAACACCCCACTACCC |
| ***FOXO1* -961/-736** upstream of TSS | CCGGCGACACTTTGTTTACT | CGTTCAGCAAAGACATCGTG |
| ***FOXO1* -9071/-8888** upstream of TSS | CAGAGTCCCTCGGTCATCTC | TGCGTTGTTGATTTTCTGCT |
